# Career perspective: John W. Severinghaus

**DOI:** 10.1186/2046-7648-2-29

**Published:** 2013-10-07

**Authors:** John W Severinghaus

**Affiliations:** 1 University of California San Francisco, San Francisco, CA 94607, USA

**Keywords:** Anesthesia, Monitor, Altitude, Acclimatization, Regulation of respiration, Cerebral blood flow, Blood gas analysis, Oxygen dissociation curve, CO_2_ electrode, Oximetry

## Abstract

After training in physics during World War II, I spent 2 years designing radar at Massachusetts Institute of Technology and then switched to biophysics. After medical school and a residency, I was doctor drafted to National Institutes of Health where I studied blood gas transport in hypothermia and developed the carbon dioxide electrode and the blood gas analyzer (pH, partial pressure of O_2_, and partial pressure of CO_2_). I joined the University of California San Francisco in 1958 in a new anesthesia department and new Cardiovascular Research Institute. My research aims were anesthesia patient monitoring, respiratory physiology, blood gas transport, and high-altitude acclimatization and pathology.

## Text

I arrived foot-first under chloroform in 1922 in Madison, WI, USA. Judged to be not academic, I built a boat, a desk I still have, radios, and hi-fi for classical music. As a Physics major during World War II, I was draft-deferred to work on radar at Massachusetts Institute of Technology. After the atom bomb was dropped, I switched to apply my knowledge on physics to medicine. During medical school (P&S 1949), I built electrophrenic respirators for several anesthesia departments [1]. Robert Dripps (University of Pennsylvania anesthesia chief) recruited me to anesthesia that needed my electronic skills. I persuaded my co-resident Peter Safar to test, on me, a small dose (20 mg) of the newly arrived succinyl choline. I had 2 min of unexpected apnea. I measured and published the uptake rate of N_2_O during anesthesia in willing patients [2]. I spent a year with Julius Comroe and Robert Forster at Pennsylvenia testing carotid chemoreceptor pharmacology and measuring lung dead space.

In 1953, to satisfy the doctor draft, I joined the US Public Health Service (USPHS) and became chief of anesthesia research at the National Institutes of Health (NIH) in Bethesda. I doubted a paper that said carbon dioxide (CO_2_) excretion was blocked during human surgical hypothermia. I disproved it by measuring blood PCO_2_ at patient temperature using Van Slyke's manometric apparatus and a homemade temperature-controlled pH analyzer. In the process, I accurately determined temperature coefficients of blood gases that became standards [3].

**Figure 1:**
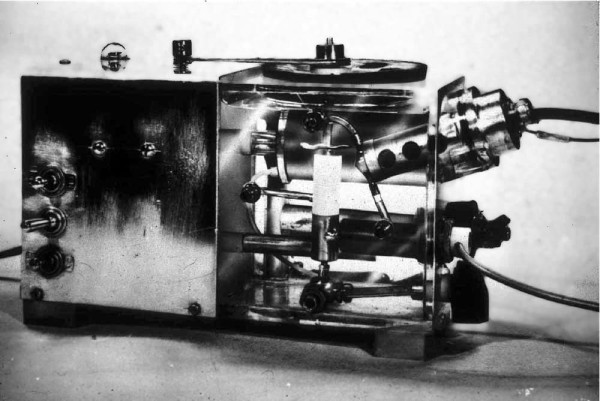
**The first blood gas analyzer.** Published as reference [4] in 1958. Copyright was by photo department at NIH and given to me in 1958. The *Journal of Applied Physiology* does not have copyright.

In 1953, at the American Physiological Society (APS) fall meeting, I heard Richard Stow (Ohio State Med) describes a PCO_2_ electrode he invented. It measured pH in a film of distilled water under a rubber membrane exposed to blood. He found its drift prevented calibration. I stabilized it by adding soda (NaHCO_3_). This Stow-Severinghaus CO_2_ electrode is now part of all blood gas analyzers. In 1957, I combined it with Leland Clark's polarographic O_2_ electrode in a thermostat bath making the first blood gas analyzer (pH, partial pressure of CO_2_ (PCO_2_), and partial pressure of O_2_ (PO_2_)) [4] (Figure 1), now in the Smithsonian museum.

I finished anesthesia residency at the University of Iowa with Stuart Cullen in 1957 (on leave from NIH). During the APS fall meeting that year, also in Iowa City, Comroe, newly appointed chair of a new University of California San Francisco (UCSF) Cardiovascular Research Institute, persuaded me to join him. I agreed instantly after he persuaded the chief of surgery at UCSF, by phone, to offer Stuart Cullen the chair of Anesthesia in a new independent (of surgery) department. Cullen and I arrived in mid-1958. I settled with family of six in my present home in Ross. I taught anesthesia (in the operating rooms) once a week, plus night call, and established labs to study respiratory physiology with Robert Mitchell and anesthesia with Ted Eger, both continuing as colleagues throughout my career.

By 1961, during a 6-week visit of Hans Loeschcke (Goettingen), Mitchell located the brain's ventral medullary cerebrospinal fluid (CSF) pH sensors, the PCO_2_ chemoreceptors [5]. This led us to study, in ourselves, the role of CSF pH and bicarbonate in acclimatization to high altitude at the UC White Mt (CA) laboratories, a range east of the Sierra Nevada [6]. We later studied the control of cerebral blood flow (CBF) at altitude, also in each other, joined by Tom Hornbein [7] soon after his ascent of Everest by the West Ridge. We repeated these acclimatization studies in Peruvian high-altitude natives at the invitation of Alberto Hurtado [8]. In 1966, Cedric Bainton and I showed that Peruvian altiplano natives have much lower (than normal) peripheral chemoreceptor (carotid body) response to hypoxia [9]. We showed that CBF was not elevated in high-altitude natives in Bolivia and fell well below normal on oxygen [10]. With many others, we tried to find the mechanisms of high-altitude pulmonary and cerebral edema. Xu and I reported increased brain tissue vascular endothelial growth factor in acutely hypoxic rats, a possible cause of capillary leakage [11].

I developed physical instrumentation useful in hypothermic anesthesia, beginning at the NIH with a battery-operated monitor of esophageal breath sounds, temperature, and electrocardiography for use with explosive anesthetics (e.g., cyclopropane) [12]. In the late 1970s, Gerry Ozanne, Bill Young, and I developed centralized monitoring of anesthetic and respiratory gas concentrations using mass spectrometry [13]. Long nylon catheters brought airway gas from the airway of patients in each of 10 (and later 20) operating rooms. The gas in them was rapidly sampled sequentially, providing data to each anesthesiologist on a computer screen about once a minute. Two firms commercially installed these systems in about 400 institutions. They became obsolete about 1995 when cheaper ‘stand-alone’ infrared capnographic type monitors were developed. After Young moved to New York, he kept watching our OR data via the Arpanet (before the Internet). One day, he phoned me, worried that in OR5 the patient's PCO_2_ was 80 Torr. The attending was shocked to be seen (in New York) just trying to get his paralyzed patient to breathe (shades of today's spying).

During a sabbatical in Copenhagen, Niels Lassen and I showed that CBF is determined by brain arteriolar, not tissue, PCO_2_. As a subject in that study, while placing a needle in my internal jugular bulb, Lassen suddenly paralyzed my right face and tongue that lasted 3 days.

I devised a blood gas slide rule for solving the effects of pH and temperature on O_2_ dissociation and on acid–base balance [14]. It was made and distributed for many years by Radiometer, the early developer of automated blood gas analyzers. To improve accuracy of the slide rule, F.J.W. Roughton from Cambridge joined Freeman Bradley (my technician for 30 years) and me using his ingenious method to very accurately measure slight desaturation at the top of the standard human oxygen dissociation curve (ODC) [15]. Our work became the standard human ODC, and I found a simple accurate equation of the ODC [16].

Other monitoring methods included the first transcutaneous combined PO_2_-PCO_2_ electrode [17]. We established a laboratory for testing the accuracy of pulse oximeters at low O_2_ saturation in volunteers, which is still in use to provide manufacturers with data for the FDA [18]. My career tended toward history when I joined Poul Astrup in writing the history of blood gases and acid–base balance [19] and then the history of pulse oximetry [20].

## Honors

I received the first American Society of Anesthesiologists (ASA) Award for Excellence in Research (1986), presented the first ASA annual John W Severinghaus Translational Science Lecture, and received an honorary degree Dr. Med. HC from the Universities of Copenhagen (1979) and Uppsala (2008).

I continue to teach UCSF residents the history and status of physical chemistry, acid–base balance, blood gas analysis, altitude acclimatization, and the discoveries of eight scientists who contributed to the discovery of oxygen and the pulmonary circulation. For the last 11 years, I have reviewed the world literature on altitude for each quarterly issue of the *Journal of High Altitude Medicine and Biology*, edited by John West.

For my other community activity, I served for 7 years on the Marin Healthcare District Board, owner of Marin General Hospital. As chair, I helped the board settle a lawsuit to recover local control. I am proud to be a liberal Democrat, an atheist Unitarian, and an active member of several medical organizations advocating Single Payer Healthcare for All. My wife Elinor and I have four children and soon will celebrate our 65th anniversary.

**Figure 2:**
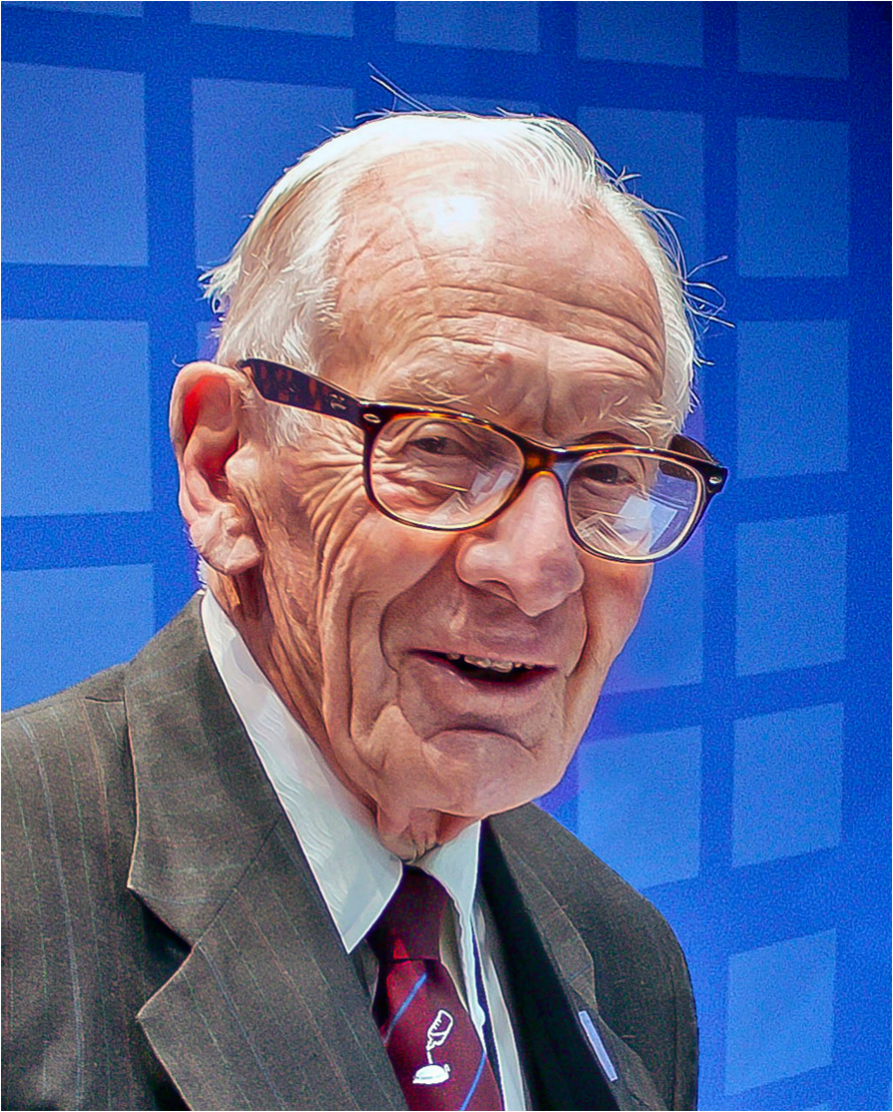
**Portrait, 2011 given to me by Sara Cheng from the University of Colorado.** No copyright.

If I started all over again and worked harder, I probably would have been less useful to the world of medicine. I loved my work. In my father's words, ‘Work is my recreation’ (Figure 2).

## Abbreviations

ASA: American Society of Anesthesiologists; APS: American Physiological Society; CBF: Cerebral blood flow; CO2: Carbon dioxide; CSF: Cerebrospinal fluid; CVRI: Cardiovascular Research Institute; NIH: National Institutes of Health; ODC: Oxygen dissociation curve; PCO2: Partial pressure of CO_2_; PO2: Partial pressure of O_2_; UCSF: University of California San Francisco; USPHS: US Public Health Service

## Competing interests

The author declares that he has no competing interests.
